# Supplementary material for: Linear Skin Defects with Multiple Congenital Anomalies (LSDMCA): An Unconventional Mitochondrial Disorder
Source: Genes (Basel). 2021 Feb 11;12(2):263. doi: 10.3390/genes12020263 (PMC7918533; doi:10.3390/genes12020263)
Supplement: Supplementary file 1 [file genes-12-00263-s001.pdf]

Supplementary Table S1. Extended clinical description of LSDMCA cases

Ref; Case Id	Mutation	Linear skin lesions	Micro-/anoph-thalmia	Corneal abnor-malities	Other eye ab-normal-ities	CNS ab-normali-ties	Intellec-tual disa-bilities	Short stat-ure	Cardiac abnor-malities	Genitouri-nary abnor-malities	Nail Dys-trophy	Diaphrag-matic hernia
[30]; case 1	X/Y translocation (Xp22.3/Yq11.2)	+	+	+	+	+	-	+	-	-	-	-
[30]; case 2	X/Y translocation (Xp22.3/Yq11.2)	+	+	+	+	+	-	+	-	+	-	-
[31]	46,X,del(X) (p22.3;pter)	+	+	+	-	+	-	-	-	-	+	-
[32]	46,X,del(X) (p22.2;pter)	+	+	+	+	-	-	NI	-	-	-	-
[33]; case 1	46,X,del(X) (p22.2;pter)	+	+	-	-	+	NA	NA	-	-	-	+
[33]; case 2	46,X,del(X) (p22.2;pter)	+	-	-	-	-	-	+	-	-	-	-
[34]; case 1	46,Xt(2;X) (p25.1;p22.1)	+	-	+	-	+	NI	NI	-	-	-	-
[34]; case 2	46,Xt(2;X) (p25.1;p22.1)	+	-	+	-	+	NI	NI	-	-	-	-
[35]	46,X,-X, + der(X)t(X,?) (p22.3;?)	+	+	+	+	+	NI	+	+	-	-	-
[36]; case 1	46,X,del(X) (p22.11;p22.31)	+	+	+	+	+	-	+	-	-	-	-
[36]; case 2	del Xp22.3-pter	+	+	+	-	+	NI	NI	+	-	+	-
[1]; case 1	46,X,del(X) (p22.2;pter)	+	+	+	+	+	+	+	-	-	-	-
[1]; case 2	46,X,del(X) (p22.2;pter)	+	-	-	+	-	-	+	-	-	-	-

Ref; Case Id	Mutation	Linear skin lesions	Micro-/anoph-thalmia	Corneal abnor-malities	Other eye ab-normal-ities	CNS ab-normali-ties	Intellec-tual disa-bilities	Short stat-ure	Cardiac abnor-malities	Genitouri-nary abnor-malities	Nail Dys-trophy	Diaphrag-matic hernia
[1];** case 3	46,X,del(X) (p22.2;pter)	NA	NA	NA	NA	+	NA	NA	NI	NI	NA	NI
[1]; case 4	Xp/Yp exchange (SRY), distal Xp monosomy	+	+	-	-	-	-	+	-	+	-	-
[37]; case 1	46,XX,t(X;Y), (p22.3;p11.2)	+	+	+	+	+	+	-	-	+	-	-
[37]; case 2	46,XX,t(X;Y), (p22.3;p11.2)	+	+	-	-	NI	NI	+	NI	-	-	-
[38]	46,X,del,(X) (p22.1)	+	+	+	+	+	+	NI	+	+	-	-
[39]	Xp/Yp exchange (SRY), distal Xp monosomy	+	+	+	+	+	+	+	+	+	-	-
[40]	Xp22.3del, cryptic Yp11.2 transl	+	+	+	-	+	NI	NI	+	+	-	-
[15]	45,X/46,X,r (X)(p22q21)/46,X, del(X)(p22)	+	+	+	+	+	-	+	-	-	+	-
[41]	46,X,der(X),t (X:Y)(p22.13;q11. 2)	-	+	+	-	-	NI	NI	+	+	-	-
[42]	46,X,der(X),t (X:Y)	+	+	-	+	-	NI	NI	NI	-	-	-
[43]	46,X,der(X),t(X:3) (p22.2; p22.2)	-	+	+	+	-	-	-	-	-	-	-
[44]	46,X,del(X) (p22.3)	-	+	-	+	-	NI	NI	NI	-	-	-

Ref; Case Id	Mutation	Linear skin lesions	Micro-/anoph-thalmia	Corneal abnor-malities	Other eye ab-normal-ities	CNS ab-normali-ties	Intellec-tual disa-bilities	Short stat-ure	Cardiac abnor-malities	Genitouri-nary abnor-malities	Nail Dys-trophy	Diaphrag-matic hernia
[45]	46,X,der(X),t (X:Y) (p22.13; p11.2)	-	+	-	-	-	NI	NI	+	-	-	-
[46]	46,X,der(X)t(X;Y)	+	+	+	-	+	-	-	+	+	-	-
[47]	46,X,del(X) (p22.3-pter)	+	+	-	-	+	+	-	-	+	-	-
[48]	46,Y,inv(X)(p22.13 ~22.2p22.32~22.3 3)[49]/46,XY[271] Mosaic	+	-	-	-	+	NI	NI	+	-	-	-
[49]; case 1	46,X,Xt(X;Y) (p22.3;p11.3)	+	+	+	+	-	-	-	+	+	-	-
[49]; case 2	46,X,Xt(X;Y) (p22.3;p11.3)	+	+	+	+	-	-	-	+	+	-	-
[50]	46,X,Xp22.3 mi-crodel	+	+	+	-	-	-	+	+	-	-	-
[51]; case 1	46,X,der(X)t(X;Y)	+	+	+	-	-	NI	-	+	+	-	-
[51]; case 2	46,X,der(X)t(X;Y)	+	+	+	-	-	NI	-	+	+	-	-
[51]; case 3	46,X,der(X)t(X;Y) (p22.3;q11)	+	+	+	+	+	NI	-	+	-	+	-
[51]; case 4	46,X,der(X)t(X;Y) (p22.3;q11.2)	-	-	+	+	-	-	+	-	-	-	-
[5]; BA 389	46,X,del(X) (p22.22)	+	+	-	+	+	+	+	-	-	-	-

Ref; Case Id	Mutation	Linear skin lesions	Micro-/anoph-thalmia	Corneal abnor-malities	Other eye ab-normal-ities	CNS ab-normali-ties	Intellec-tual disa-bilities	Short stat-ure	Cardiac abnor-malities	Genitouri-nary abnor-malities	Nail Dys-trophy	Diaphrag-matic hernia
[5]; BA 644	46,X,der(X),t (X:Y)(p22.22; q11.2)	+	+	-	+	NI	NI	+	-	-	-	-
[5]; BA 659	46,X,der(X),t (X:Y) (p22.22;q25.3)	+	+	+	-	NI	+	-	-	-	-	-
[5]; BA 745	46,X,del(X) (p22.22,p22.22)	+	+	+	-	-	NI	NI	-	-	-	-
[52]; ***	46,X,der(X)t (X;Y)(p22.2;q11.2)	+	+	+	+	+	NA	NA	+	+	-	-
[53]	46,X,del(X) (p22.2)	+	-	+	+	-	-	+	-	-	-	-
[54]	46,X,del(X) (p22.2;pter)	+	+	+	+	-	+	+	-	+	-	-
[55]; ***	46,X,del(X) (p22.3)(3,6Mb)	+	+	-	-	+	NA	NA	+	+	-	+
[56]	Xp22.3 ab	+	+	-	-	+	NI	NI	+	-	-	-
[57]	46,X,del(X) (p22.2;pter)	+	+	+	-	+	+	+	-	+	-	+
[58]; case III.3	46,X,del(X) (p22.2)	-	+	+	-	+	-	+	+	-	-	-
[58]; case II.1	46,X,del(X) (p22.2)	-	-	-	-	-	-	-	-	-	-	-
[59]; pro-band	46,X,del(X) 12.9Mb ter del	+	+	+	+	+	+ Autis-tic be-haviors	+	-	-	NI	-

Ref; Case Id	Mutation	Linear skin lesions	Micro-/anoph-thalmia	Corneal abnor-malities	Other eye ab-normal-ities	CNS ab-normali-ties	Intellec-tual disa-bilities	Short stat-ure	Cardiac abnor-malities	Genitouri-nary abnor-malities	Nail Dys-trophy	Diaphrag-matic hernia
[59]; Mother	46,X,del(X) 12.9Mb ter del	-	-	+	-	-	-	+	-	-	NI	-
[60]; pro-band	46,X,del(X)(p22.2) 11.5Mb ter del	+	+	+	+	-	-	NI	+	-	+	-
[60]; Mother	46,X,del(X)(p22.2) 11.5Mb ter del	-	-	-	-	-	-	-	-	-	-	-
[29]; pro-band	46,X,del(X)(p22.2) int del ~220 kb in-cluding *HCCS*	-	+	+	+	NI	-	-	-	-	NI	-
[29]; mother	46,X,del(X)(p22.2) int del ~220 kb in-cluding *HCCS*	+	+	-	+	NI	-	-	-	-	NI	-
[13]; Patient 1; III.1	46,X,del(X)(p22.2p 22.2), int del ~850 kb including *HCCS*	-	+	+	+	+	-	-	-	-	-	-
[13]; Patient 1; II.1	46,X,del(X)(p22.2p 22.2), int del ~850 kb including *HCCS*	-	-	-	-	-	-	-	-	-	-	-
[13]; Patient 1; II.2	46,X,del(X)(p22.2p 22.2), int del ~850 kb including *HCCS*	-	-	-	-	-	-	-	-	-	-	-
[13]; Patient 4	46,X,del(X)(p22)	+	+	+	+	+	+	+	-	-	-	-
[13]; Patient 5	46,X,del(X)(p22)	+	+	+	-	-	+	-	-	+	-	-

Ref; Case Id	Mutation	Linear skin lesions	Micro-/anoph-thalmia	Corneal abnor-malities	Other eye ab-normal-ities	CNS ab-normali-ties	Intellec-tual disa-bilities	Short stat-ure	Cardiac abnor-malities	Genitouri-nary abnor-malities	Nail Dys-trophy	Diaphrag-matic hernia
[13]; Patient 6	46,X,del(X)(p22) int del ≥3 Mb in-cluding *HCCS*	+	+	+	-	-	-	-	-	-	-	-
[61]	46,XX,ish del(p22.2p22.31) 3,3 Mb del mosaic	+	+	-	-	-	NI	-	+	NI	-	-
[62]	46,X,del(X)(p22)	+	+	+	+	-	-	-	-	+	+	-
[62]; mother	46,X,del(X)(p22)	+	-	-	-	NI	NI	NI	NI	NI	NI	NI
[62]; elder daugh-ter	46,X,del(X)(p22)	+	-	-	-	NI	NI	NI	NI	NI	NI	NI
[63]	46,X,del(X)(p22.33 p22.2) 11Mb del	+	+	-	-	+	-	NI	+	-	-	+
[64]	46,XX,del(X)(p22. 3p22.2) 11.5Mb del	+	+	+	-	+	+	+	-	+	-	-
[66]	Xp22.2 ter del	+	+	+	+	-	-	-	+	+	-	+
[67]; case 1	Xp22 del	+	+	+	-	-	NI	NI	-	+	-	-
[67]; case 2	46,X,der(X)t (X;Y)	+	+	+	-	+	NI	NI	+	+	-	-
[2]; case II.7	8.6kb del spanning 5'*HCCS* and *MID1*	+	+	+	-	-	-	-	+	-	-	-
[2]; case II.1	8.6kb del spanning 5' *HCCS* and *MID1*	-	-	-	+	-	-	-	-	-	-	-

Ref; Case Id	Mutation	Linear skin lesions	Micro-/anoph-thalmia	Corneal abnor-malities	Other eye ab-normal-ities	CNS ab-normali-ties	Intellec-tual disa-bilities	Short stat-ure	Cardiac abnor-malities	Genitouri-nary abnor-malities	Nail Dys-trophy	Diaphrag-matic hernia
[2]; case II.3	8.6kb del spanning 5’ *HCCS* and *MID1*	-	+	-	-	NI	NA	NA	-	-	-	+
[2]; case I.1	8.6kb del spanning 5’ *HCCS* and *MID1*	-	-	-	-	-	-	-	-	-	-	-
[2]; case MS1	point mut *HCCS*	+	+	+	+	+	+	-	-	-	-	-
[2]; case MS2	point mut *HCCS*	-	+	+	-	+	+	-	+	-	-	-
[14]	point mut *HCCS*	-	+	+	-	-	-	-	-	-	-	-
[13]; Patient 2	point mut *HCCS*	+	+	+	+	+	NA	+	+	-	-	-
[13]; Patient 3	point mut *HCCS*	-	+	+	+	NI	+[a]	-	-	-	-	-
	**TOT # cases**	**56/75**	**59/75**	**50/75**	**34/75**	**40/70**	**16/50**	**24/54**	**25/70**	**21/72**	**6/69**	**5/73**
	**%**	**75**	**79**	**67**	**45**	**57**	**32**	**44**	**36**	**29**	**9**	**7**
[3]; case 1	point mut *COX7B*	+	-	-	-	+	-	+	-	-	+	-
[3]; case 2	point mut *COX7B*	+	-	-	-	+	+	+	+	+	-	+
[3]; case I.2	point mut *COX7B*	+	-	-	+	+	+	-	+	-	-	-

Ref; Case Id	Mutation	Linear skin lesions	Micro-/anoph-thalmia	Corneal abnor-malities	Other eye ab-normal-ities	CNS ab-normali-ties	Intellec-tual disa-bilities	Short stat-ure	Cardiac abnor-malities	Genitouri-nary abnor-malities	Nail Dys-trophy	Diaphrag-matic hernia
[3]; case II.4	point mut *COX7B*	+	-	-	+	+	+[b]	-	-	-	-	-
	**TOT # cases**	**4/4**	**0/4**	**0/4**	**2/4**	**4/4**	**3/4**	**2/4**	**2/4**	**1/4**	**1/4**	**1/4**
	**%**	**100**	**0**	**0**	**50**	**100**	**75**	**50**	**50**	**25**	**25**	**25**
[4]; subject 1	point mut *NDUFB11*	+	-	-	+	-	-	NA	+	-	-	-
[4]; subject 2	point mut *NDUFB11*	+	-	-	+	+	+	+	+	-	-	-
[4]; mother subject 2	point mut *NDUFB11*	-	-	-	-	-	-	-	-	-	-	-
[4]; ****		NA	-	-	-	+	NA	NA	+	NI	NA	NI
	**TOT # cases**	**2/3**	**0/3**	**0/3**	**2/3**	**1/3**	**1/3**	**1/2**	**2/3**	**0/3**	**0/3**	**0/3**
	**%**	**67**	**0**	**0**	**67**	**33**	**33**	**50**	**67**	**0**	**0**	**0**

**This patient was an aborted fetus with exencephaly and absence of the entire scalp skin. ***Malformed female infants died 9hs [55] and 5 days [52] after delivery. ****Aborted female fetus from the mother of subject 2. These cases were not considered in the total number of patients for the calculation of the percentage of occurrence of clinical signs as well as all the other patients for which that specific feature had not been studied (NI) or could not be observed (NA) because the patient was too young and so on.

NI, not investigated
NA, not applicable

[a]mild motor delay; [b]attention deficit disorders (ADHD)

Other Eye abnormalities: prolapsed iris, severe myopia, orbital cyst, hypopigmented and disorganized retinal pigmented epithelium, cataracts, choroidal thickening, coloboma, chorioretinopathy, glaucoma, lens abnormalities, aniridia, pale optic disk and altered visual-evoked potential.

CNS abnormalities: microcephaly, agenesis of the corpus callosum, colpocephaly, seizures, hydrocephalus, ventriculomegaly, cystic cerebral malformation.

Cardiac abnormalities: atrial and ventricular septal defect, atrio-ventricular block, histiocytoid cardiomyopathy, supraventricular tachycardia, junctional ectopic tachycardia, murmur, coarctation of the aorta, aortic stenosis/atresia and patent foramen ovale.

Genitourinary abnormalities: hypospadias, intersexual genitalia, hypoplastic genitalia, imperforate or displaced anus and polycystic ovary syndrome.

Abbreviations: Ref, reference; del, deletion; microdel, microdeletion; ter, terminal; int, interstitial; ab, abnormalities; ADHD attention deficit disorders.

Note: all references were cited in the main text and the number of the references refer to the reference list of the main text.
